# Perceptions of ethical decision-making climate among clinicians working in European and US ICUs: differences between religious and non-religious healthcare professionals

**DOI:** 10.1186/s12910-025-01178-5

**Published:** 2025-02-05

**Authors:** Hanne Irene Jensen, Hans-Henrik Bülow, Lucas Dierickx, Stijn Vansteelandt, Rosanna Vaschetto, Gábor Élö, Ruth Piers, Dominique D. Benoit

**Affiliations:** 1 Departments of Anaesthesiology and Intensive Care, Vejle Hospital, University Hospital of Southern Denmark, Beriderbakken 4, Vejle, 7100 Denmark; 2 Department of Anaesthesiology and Intensive Care, Kolding Hospital, University Hospital of Southern Denmark, Sygehusvej 24, Kolding, 6000 Denmark; 3 Institute of Regional Health Research, University of Southern Denmark, J.B.Winsløwsvej 19, Odense, 5000 Denmark; 4 Department of Anaesthesiology and Intensive Care, Holbaek Hospital, Smedelundsgade 60, Holbaek, 4300 Denmark; 5 Department of Marketing, Innovation and Organisation, Ghent University, Tweekerkenstraat 2, Ghent, 9000 Belgium; 6 Department of Applied Mathematics, Computer Science and Statistics, Ghent University, Krijgslaan 281, S9, Ghent, 9000 Belgium; 7 University of Eastern Piedmont, Via Solaroli 17, Novara, 28100 Italy; 8 Semmelweis University, Üllői út 26, Budapest, 1085 Hungary; 9 Department of Geriatrics, Ghent University Hospital and Ghent University, Corneel Heymanslaan 10, Ghent, 9000 Belgium; 10 Department of Intensive Care Medicine, Ghent University Hospital and Ghent University, Corneel Heymanslaan 10, Ghent, 9000 Belgium

**Keywords:** Conflicts, Decision-making, End-of-life, Ethical climate, Intensive care unit, Inter-professional collaboration, Religion, Teamwork

## Abstract

**Background:**

Making appropriate end-of-life decisions in the intensive care unit (ICU) requires shared interprofessional decision-making. Thus, a decision-making climate that values the contributions of all team members, addresses diverse opinions and seeks consensus among team members is necessary. Little is known about religion’s influence on ethical decision-making climates. Therefore, this study aimed to examine the association between religious belief and ethical decision-making climates.

**Methods:**

The study was a cross-sectional analytical observation study as a part of the prospective observational DISPROPRICUS study. A total of 2,275 nurses and 717 physicians from 68 ICUs representing 12 countries in Europe and the US participated. All participants were asked which religion (if any) they belonged to and how important their religion (if any) was for their professional attitude towards end-of-life care. Perceptions of ethical decision-making climates were evaluated using a validated, 35-item self-assessment questionnaire that evaluates seven factors. Using cluster analysis, ICUs were categorised into four ethical decision-making climates: good, average (with nurses’ involvement at the end of life), average (without nurses’ involvement at the end of life) and poor.

**Results:**

Of the 2,992 participants, 453 (15%) were religious (had religious convictions and found them important or very important for their attitude towards end-of-life care). The remaining 2,539 were non-religious (i.e. had religious convictions but assessed that they were not important for their attitude towards end-of-life care). When adjusting for country and ICU, the overall perception of the four ethical climates was associated with religious beliefs, with non-religious healthcare providers having more positive perceptions of the ethical climates compared to religious healthcare providers (*p* < 0.01). Within good climates, non-religious healthcare providers rated leadership by physicians (*p* < 0.01), interdisciplinary reflection (*p* *=* 0.049) and active decision-making by physicians (*p* *=* 0.02) as more positive compared to religious participants. In poor climates, religious healthcare providers had a more positive perception of the active involvement of nurses (*p* = 0.01). Within the other climates, no differences were found.

**Conclusions:**

Overall perceptions of ethical decision-making climates were associated with religious beliefs, with non-religious healthcare providers generally having a more positive perception of the ethical climates than religious healthcare providers.

**Supplementary Information:**

The online version contains supplementary material available at 10.1186/s12910-025-01178-5.

## Background

Making the most appropriate end-of-life decisions in the ICU requires shared, interprofessional decision-making [1]. To foster this requirement, a decision-making climate that values the contributions of all team members, addresses diverse opinions and seeks consensus among team members is necessary [1]. Poor ethical decision-making climates (EDM-Cs) may lead to suboptimal decision-making processes and the provision of excessive care [2]. Likewise, a poor EDM-C may entail team conflicts, poor family support, moral distress, burnout and intent to leave [1, 3].

A good decision-making climate requires interdisciplinary communication and collaboration [1, 4], empowered by physicians [5], and an ethical working environment that facilitates the possibility of ethical debate, includes nurses in decision-making and tolerates different opinions and values [6]. Nurses have been found to consistently perceive ethical climates in ICUs as worse compared to physicians across different climates [7–9], and nurses are prone to leave their ICU jobs if they experience moral distress at work [9, 10].

There are differences among the world’s major religions when it comes to decision-making in the ICU, especially concerning the withdrawal of life-sustaining treatment [11]. This may lead to conflicts between religious believers and nonbelievers or conflicts between different religious groups when decisions must be made. Among religious physicians, 15–30% will not follow a competent patient’s wish to abstain from treatment [12], and an analysis of 112 standardised, simulated ICU family meetings found that the self-reported religiosity of intensivists was associated with increased odds of perceived conflicts during the simulated meeting with an actor portraying a religious family surrogate [13].

Little is known about the influence of religion on EDM-Cs. Therefore, the aim of the current study was to examine the association between religious beliefs and EDM-Cs. The hypothesis was that non-religious healthcare providers (HCPs) would rate their EDM-Cs higher than religious HCPs would rate their EDM-Cs.

## Methods

This was a cross-sectional analytical observation sub-study of the DISPROPRICUS study, which examined the possible association between EDM-Cs and prognostic values (patient outcomes) when nurses and physicians in a 28-day period perceived the care provided to be excessive [2]. ICUs were selected via the Ethics Section of the European Society of Intensive Care Medicine, from the APPROPRICUS study group [14] and via contact with experts in communication and end-of-life (EOL) care. From April–May 2014, personal data– including profession, rank, sex, age, ICU experience, religious beliefs and importance of these beliefs together with perceptions of EDM-Cs were evaluated among clinicians from 68 adult ICUs in 12 European countries (Belgium, the Czech Republic, Denmark, France, Germany, Greece, Hungary, Italy, Portugal, the United Kingdom, Sweden and the Netherlands) and the United States (US).

The tool used was the validated, self-assessed Ethical Decision-Making Climate Questionnaire (EDMCQ; [6]), which is based on the ICU Safety Attitude Questionnaire [15], the Leader Behavior Description Questionnaire (LBDQ; [16, 17]), the Interprofessional Practice and Education Quality Scale (IPEQS; [16, 18]) and the questionnaire used in the APPROPRICUS study [14]. The theoretical framework behind the EDMCQ and the EDMCQ tool were developed through a modified Delphi method [6]. It encapsulated three key domains of ethical decision-making (EDM) within the healthcare context: interdisciplinary collaboration and communication; leadership by physicians; and the ethical environment. The EDMCQ was subsequently validated using both exploratory and confirmatory factor analyses [6]. Additionally, measurement invariance was assessed to ensure that the variables utilised in the analysis represented comparable constructs across diverse groups [6]. In addition to the EDMCQ, questions regarding HCP characteristic were included. These questions were based on a former questionnaire [14] and tested for face and content validity by participating HCPs from different countries. The study questionnaire was first presented as supplementary material in [2], but can also be found as supplementary material in this paper (Table S1). The questionnaire was developed in English and subsequently scientifically two-way translated into the languages of the participating countries. All HCPs at the participating ICUs were asked to fill in the questionnaire which was available electronically on the DISPROPRICUS study website to which each HCP received a personal log-in.

### Religious beliefs

The two questions regarding religion were: (1) “What is your religion?” with response options: “Roman Catholic, Protestant, Greek-Orthodox, Muslim, Jewish, Buddhist, Non-religious, I do not wish to answer this question”, and (2) “How important is your religion for your professional attitude towards your professional end-of-life decisions?” with response options: “Not important, Not very important, Important, Very important”. For analysis, the data containing information on HCPs were split into data on religious HCPs (those rating the importance of their religion as important or very important in their attitude towards EOL care) and 2) non-religious HCPs (those identifying as non-religious as well as those identifying with a specific religion but rating the importance of their religion as not very important or not important in their attitude towards EOL care).

### Ethical decision-making climates

Developed using explorative and confirmatory factor analyses [6], the final model of EDM-Cs included seven meaningful factors (F): F1. self-reflective and empowering leadership by physicians, F2. a practice and culture of open interdisciplinary reflection, F3. a culture of not avoiding EOL decisions, F4. a culture of mutual respect within the interdisciplinary team, F5. active involvement of nurses in EOL care and decision-making, F6. active decision-making by physicians and F7. a practice and culture of ethical awareness. The questions included in each factor [7] and the full questionnaire with response options and factor analyses [2] have previously been published.

To identify possible types of ethical climates within all participating ICUs, a dimension reduction was carried out by means of cluster analysis using the seven factors identified [2]. Each ICU provided responses from several clinicians, each with their own perception of that ICU’s ethical climate. The average score across HCPs for each factor in a given ICU was calculated and used as input for the cluster analysis at the ICU level. The cluster analyses yielded four grades of EDM-Cs: good, average (with involvement of nurses at the end of life), average (without involvement of nurses at the end of life) and poor. Good climates are characterised by active leadership among senior clinicians and mutual respect, which enables interdisciplinary reflection and decision-making overall. HCPs in average (+) climates still had a positive perception of their EDM-C but reported lower scores on average for each of the factors. In comparison to the average (+) climates, there was a negative perception of how actively nurses are involved in EOL care and decision-making in the average (-) climates. Finally, HCPs in poor climates perceived a need to improve in each of the seven factors [2].

### Data analysis

Non-religious and religious HCPs were classified into the four EDM-C groups. To acknowledge correlations between measurements within ICUs and to adjust for the confounding effects of cultural differences not related to religion, mixed models adjusting for country as a fixed effect and ICU as a random effect were used to compare the overall perceptions of the four climates.

Furthermore, differences between religious and non-religious HCPs in perceptions of the EDM-Cs were assessed by comparing median factor scores for each of the seven EDM-C factors using adjusted-median mixed models with country as a fixed effect and ICU as a random effect. This comparison was first made with the entire sample of HCPs and subsequently within each of the four ethical climates separately. A *p*-value < 0.05 was considered significant.

## Results

A total of 717 physicians and 2,275 nurses participated in the study, representing 68 ICUs from 12 European countries and the US. The overall response rate for the questionnaire was 63%; for physicians, it was 61%, and for nurses, 63%.

A total of 1,802 HCPs stated that they had a religious conviction or ticked ‘Do not want to answer’. Two-thirds were either Roman Catholics or Protestants. In supplementary table S2, percentages for each country are presented. Of those who had religious convictions, the majority found that these beliefs were not or were not very important for their attitude towards EOL care. Within religions, Greek Orthodox Christians and Muslims were likeliest to find that their religion was important or very important for their attitude towards EOL care (Table 1).

**Table 1:** Participants’ religious beliefs

	Total (*n* = 2,992)	
Religious conviction^1^ (yes), n %)	1,802	(60)
Of which:		
Buddhist	10	(1)
Greek-Orthodox	179	(10)
Jewish	9	(1)
Muslim	30	(2)
Protestant	534	(30)
Roman Catholic	687	(38)
Other	162	(10)
”Do not wish to answer”	191	(11)
How important is religious conviction in your attitude towards end-of-life decisions^2,^ n (%)		
Not important	849	(47)
Not very important	500	(28)
Important	317	(18)
Very important	136	(8)
Religious conviction is important to very important in your attitude towards end-of-life (yes)^3^, n (%)		
Buddhist	3	(30)
Greek-Orthodox	83	(46)
Jewish	1	(11)
Muslim	14	(47)
Protestant	120	(22)
Roman Catholic	156	(23)
Other	54	(33)
“I do not wish to answer”	22	(12)

1. The 1,802 who stated they had a religious conviction or did not wish to answer

2. Percentages based on the 1,802 who stated they had a religious conviction or did not wish to answer

3. Percentages based on the total number of participants with a religious conviction within each religion (numbers presented at top of table)

Of the 2,992 participating HCPs, 453 were considered religious (had religious convictions and found them important or very important for their attitude towards EOL care). The remaining 2,539 were considered non-religious (including those who had religious convictions but assessed that they was not important for their attitude towards EOL decisions) (Table 2).

### Association between religious beliefs and end-of-life ethical decision-making climates

When adjusting for country and ICU, the overall perceptions of the EDM-Cs were associated with religious beliefs, with non-religious HCPs having a more positive perception of their ethical climates compared to non-religious HCPs (*p* < 0.01) (Table 2).

**Table 2:** Healthcare provider characteristics

	Non-religious HCP^1^		Religious HCP^1^		Adjusted *p*-value^2^
	*n* = 2,539		*n* = 453		
Age, median(IQR)	38	(30–47)	40	(32–48)	< 0.001
Gender (Female), n(%)	1,802	(71)	332	(73)	0.36
Have a partner, n(%)	1,963	(77)	337	(74)	0.56
Have children, n(%)	1,461	(58)	293	(65)	< 0.001
Role, n(%)					< 0.01
Nurse	1,921	(76)	354	(78)	
Junior physician	269	(10)	39	(9)	
Senior physician	349	(14)	60	(13)	
Years working in ICU, median(IQR)	7	(3–16)	9	(4–16)	< 0.001
Weekly working hours, median(IQR)	37	(32–40)	40	(36–40)	0.09
Nightshifts, n(%)	2,171	(86)	371	(82)	0.05
Ethical Climate, n(%)					< 0.01
Good	478	(18.8)	57	(12.6)	
Average with participation of nurses	1,077	(42.4)	176	(38.8)	
Average without participation of nurses	217	(8.6)	85	(18.8)	
Poor	767	(30.2)	135	(29.8)	

1. HCP: Healthcare provider

3. Mixed models adjusting for country as a fixed effect and ICU as a random effect

Within climates, non-religious HCPs rated leadership by physicians (adjusted difference in medians: 0.24, *p* < 0.01), interdisciplinary reflection (adjusted difference in medians: 0.11, *p* *=* 0.049) and active decision-making by physicians (adjusted difference in medians: 0.16, *p* *=* 0.02) as more positive compared to religious HCPs. In both average climates (with and without the involvement of nurses in EOL decisions), no significant differences were found in the perceptions of any of the seven EDM-C factors. In poor climates, religious HCPs had a more positive perception of the active involvement of nurses (adjusted difference in medians: 0.20, *p* = 0.01) (Table 3).

**Table 3:** Perceptions per climate

**Factor**	Non-religious healthcare providers (*n* = 2539)		Religious healthcare providers (*n* = 453)						
	median	IQR	median	IQR	diff	p	adjusted median diff	adjusted p^1^	highest median after adjustment
**Good climate**									
F1. Leadership by physicians	0.55	(-0.11;1.02)	0.59	(-0.21;1.34)	0.04	0.82	0.24	< 0.01**	non-religious
F2. Interdisciplinary reflection	0.77	(0.22;1.25)	0.68	(-0.08;1.20)	0.09	0.44	0.11	0.049*	non-religious
F3. Culture of not avoiding EOL-DM	0.26	(-0.37;0.99)	0.18	(-0.58;0.71)	0.08	0.71	0.14	0.29	non-religious
F4. Mutual respect	0.75	(0.25;1.40)	0.41	(-0.09;1.35)	0.34	0.03*	0.05	0.37	non-religious
F5. Active involvement nurses	0.45	(-0.04;1.10)	0.56	(0.17;1.34)	0.11	0.39	0.09	0.51	non-religious
F6. Active DM physicians	0.55	(0.15;0.91)	0.45	(-0.29;0.89)	0.1	0.38	0.16	0.02*	non-religious
F7. Ethical awareness	0.53	(0.04;1.34)	0.21	(-0.14;0.69)	0.32	< 0.001***	0.10	0.35	non-religious
**Average with nurses’ involvement at EOL**									
F1. Leadership by physicians	0.13	(-0.44;0.67)	0.21	(-0.43;0.77)	0.08	0.31	0.05	0.49	non-religious
F2. Interdisciplinary reflection	0.13	(-0.43;0.62)	0.25	(-0.40;0.72)	0.12	0.15	0.08	0.41	religious
F3. Culture of not avoiding EOL-DM	0.33	(-0.30;0.81)	0.26	(-0.51;0.76)	0.07	0.45	0.002	0.97	religious
F4. Mutual respect	0.19	(-0.24;0.42)	0.22	(-0.29;0.45)	0.03	0.35	0.04	0.30	religious
F5. Active involvement nurses	0.35	(-0.02;0.73)	0.33	(-0.09;0.65)	0.02	0.57	0.05	0.36	non-religious
F6. Active DM physicians	0.19	(-0.42;0.53)	0.13	(-0.44;0.48)	0.06	0.4	0.01	0.94	non-religious
F7. Ethical awareness	0.11	(-0.21;0.43)	0.1	(-0.29;0.32)	0.01	0.82	0.04	0.16	religious
**Average without nurses’ involvement at EOL**									
F1. Leadership by physicians	0.37	(-0.46;0.92)	0.58	(-0.28;1.07)	0.21	0.25	0.01	0.98	non-religious
F2. Interdisciplinary reflection	0.32	(-0.45;0.88)	0.21	(-0.38;0.84)	0.11	0.43	0.09	0.62	non-religious
F3. Culture of not avoiding EOL-DM	0.07	(-0.68;0.63)	-0.13	(-0.54;0.73)	0.2	0.24	0.06	0.67	religious
F4. Mutual respect	0.02	(-0.61;0.48)	0.07	(-0.69;0.38)	0.05	0.71	0.03	0.85	non-religious
F5. Active involvement nurses	-0.8	(-1.42;-0.28)	-0.94	(-1.54;-0.38)	0.14	0.36	0.02	0.84	religious
F6. Active DM physicians	0.5	(0.11;0.95)	0.64	(0.13;1.11)	0.14	0.18	0.06	0.66	religious
F7. Ethical awareness	-0.08	(-0.75;0.32)	-0.2	(-0.62;0.22)	0.12	0.29	0.09	0.49	non-religious
**Poor**									
F1. Leadership by physicians	-0.46	(-1.19;0.27)	-0.14	(-1.08;0.64)	0.32	0.02*	0.17	0.19	religious
F2. Interdisciplinary reflection	-0.51	(-1.31;0.17)	-0.47	(-1.20;0.18)	0.04	0.74	0.09	0.53	religious
F3. Culture of not avoiding EOL-DM	-0.47	(-1.09;0.15)	-0.48	(-0.93;0.09)	0.01	1	0.18	0.07	religious
F4. Mutual respect	-0.33	(-1.08;0.17)	-0.3	(-1.24;0.20)	0.03	0.84	0.03	0.80	religious
F5. Active involvement nurses	-0.42	(-1.10;0.23)	-0.54	(-1.26;0.03)	0.12	0.38	0.20	0.01*	religious
F6. Active DM physicians	-0.39	(-1.17;0.29)	-0.21	(-1.11;0.34)	0.18	0.22	0.09	0.35	religious
F7. Ethical awareness	-0.24	(-0.89;0.10)	-0.36	(-1.20;0.02)	0.12	0.26	0.04	0.65	religious

EOL-DM: End-of-life decision-making, DM: Decision-making.

1. Mixed model adjusting for country as a fixed effect and ICU as a random effect

* Significant at < 0.05 level

** Significant at < 0.01 level

*** Significant at < 0.001 level

When looking at the seven factors constituting EDM, no significant differences between religious and non-religious HCPs were found after adjusting for country and ICU (see the Supplementary Material, Table S3).

## Discussion

After adjusting for country and ICU, the overall ethical climate ratings were associated with religious beliefs, with non-religious HCPs having a more positive perception of their ethical climates compared to religious HCPs. Within good climates, non-religious HCPs rated leadership by physicians, interdisciplinary reflection and active decision-making by physicians as more positive compared to religious HCPs. In poor climates, religious HCPs had a more positive perception of the active involvement of nurses in EOL care and decision-making. However, no significant differences were found between religious and non-religious HCPs when looking at the seven factors that constitute EDM-Cs.

In a time of rapid globalisation and growing cultural diversity, it is inevitable that clashes between belief systems will occur. In London, one-third of the population was born outside the UK, and over 300 languages are spoken in the capital [19]. This may lead to large variations in perceptions of both how work in the ICU should be conducted and what roles ICU nurses should play [20]. Many physicians from non-Western regions may be less experienced in using and working within the concepts of medical utility and futility. This may lead to prolonged ICU stays [21] and less-qualified communication [22] and, through this, have a negative influence on EDM-Cs. Bülow et al. found significant differences in EOL decisions between actively religious doctors, nurses, patients and families and those who identified themselves as merely affiliated with a religion. Religious respondents requested more treatment, were more in favour of prolonging life and were less likely to want euthanasia than those only affiliated with a religion [12]. These attitudes towards one’s own treatment are quite likely to be reflected in the decisions HCPs make on behalf of the patients. Another study discusses the possibility that religious intensivists whose religious traditions dictate a specific approach to EOL care face the possibility that a patient or surrogate (or colleague) will make a choice that conflicts with their physician’s personal values [13]. Secular intensivists may be more comfortable supporting a broader range of approaches to EOL care, placing them at a lower risk of experiencing this conflict and moral distress [13]. The current study indicates an association between religion and evaluation of EDM-Cs, suggesting that, to promote a good EDM-C, it is important that there be an openness towards religion-based opinions, and ICU leadership must acknowledge diversities based on religion.

Although the current study found a significant association between the religions of HCPs and their overall perceptions of the four EDM-Cs, the adjusted median differences in the seven factors constituting the ethical climates were not statistically significant. When comparing nurses’ and physicians’ perceptions of their EDM-Cs (in another sub-study of the DISPROPRICUS study), substantially larger and statistically significant differences were found [7], suggesting that religion plays a role in connection with EDM-Cs but is not as dominant as other factors, such as profession.

The current study also indicates that non-religious HCPs in good climates perceive leadership among clinicians as more empowering and self-reflective compared to religious HCPs. This may suggest that discussions and reflections may be better fostered in ICUs dominated by non-religious HCPs, where the hierarchy may be less dominant, and the leader can be questioned without the HCPs being afraid of a reprimand. Likewise, the study indicates that differences of opinions are better tolerated in good climates.

The strengths of this study include the substantial number of multinational participants, inclusion of both physicians and nurses, and identification of factors and climates based on previously published comprehensive statistical analyses.

The study also has several limitations. The data came only from the Western hemisphere and are therefore only directly generalisable to Western countries. Participation from Asia, Middle East and Africa may have altered the findings and the conclusions, as seen in the ETHICUS-2 study [23]. The participating ICUs were included via network contacts, which may have introduced selection bias into the results. Furthermore, the effects of religion may be difficult to disentangle from the effects of cultural factors; although our results were adjusted for country, there may well be residual confounding. The analyses were based on the dichotomy of being religious or non-religious, which, in real life, probably includes more diverse variations, and identification with a specific faith may individually and culturally have different impacts in Europe and the US on conceptions of level of religiosity and on decision-making. Furthermore, the study only looked at all the main religions, but did not include a broader perception of spirituality. A final and important limitation is that no distinction was made between different religious affiliations. This study examined differences between the importance of religion for HCPs but did not contain distinctions between specific religions and their differing views on the EOL. Two literature reviews have found that religions have differing views on euthanasia and do-not-resuscitate orders [11, 24] and on what constitutes disproportionate treatment [24]. This study thus highlights that further research on religion in an ICU and EOL decision-making context should be concerned with specific religions and their differing values regarding the EOL.

## Conclusion

Overall, ethical climate was associated with religious belief, with non-religious healthcare provides generally having a more positive perception of their ethical climate compared to religious healthcare providers.

## Electronic supplementary material

Below is the link to the electronic supplementary material.

Supplementary Material 1

Supplementary Material 2

Supplementary Material 3

## Data Availability

The datasets used and/or analysed during the current study are available from the corresponding author on reasonable request.
