# Supplementary material for: Perceptions of ethical decision-making climate among clinicians working in European and US ICUs: differences between religious and non-religious healthcare professionals
Source: BMC Med Ethics. 2025 Feb 5;26:21. doi: 10.1186/s12910-025-01178-5 (PMC11796059; doi:10.1186/s12910-025-01178-5)
Supplement: Supplementary file 1 — Supplementary Material 1 [file 12910_2025_1178_MOESM1_ESM.docx]

**Table S1. Percentages of different religions for each participating country: Study data and general country data^1^**

|  | Protestant | Roman Catholic | Greek-Orthodox | Muslim | Buddhist | Jewish | Other | Non-religious | Do not wish to answer |
| --- | --- | --- | --- | --- | --- | --- | --- | --- | --- |
| Belgium (n=526) |  |  |  |  |  |  |  |  |  |
| Study | 0.4 | 40 | 0.6 | 1 | 0.4 |  | 2 | 48 | 8 |
| Country | 3 | 54 | 1 | 6 | 0.2 | 0.3 | 2 | 31 |  |
| Czech Republic (n=353) |  |  |  |  |  |  |  |  |  |
| Study | 1 | 22 | 0.3 |  | 0.6 |  | 4 | 58 | 14 |
| Country | 3 | 27 |  | 0.5 |  | < 0.1 |  | 60 |  |
| Denmark (n=238) |  |  |  |  |  |  |  |  |  |
| Study | 67 | 2 |  |  | 0.4 |  | 6 | 21 | 4 |
| Country | 74 | 0.1 |  | 5 | 0.6 | 0.1 | 2 | 18 |  |
| France (n=138) |  |  |  |  |  |  |  |  |  |
| Study | 0.7 | 42 |  | 3 | 0.7 | 0.7 | 1 | 40 | 12 |
| Country | 6 | 55 | 4 | 8 | 1 | 1 | 1 | 30 |  |
| Germany (n=88) |  |  |  |  |  |  |  |  |  |
| Study | 30 | 35 | 2 |  | 1 |  | 7 | 22 | 3 |
| Country | 26 | 28 | 2 | 5 |  | 0.1 | 2 | 40 |  |
| Greece (n=176) |  |  |  |  |  |  |  |  |  |
| Study |  |  | 94 |  |  |  | 0.6 | 2 | 3 |
| Country |  | 1 | 95 | 1 |  | < 0.1 |  |  |  |
| Hungary (n=72) |  |  |  |  |  |  |  |  |  |
| Study | 28 | 49 | 1 |  |  | 1 | 4 | 10 | 7 |
| Country | 25 | 68 | 0.5 |  |  | 0.1 |  | 5 |  |
| Italy (n=73) |  |  |  |  |  |  |  |  |  |
| Study |  | 77 | 1 |  |  |  | 4 | 16 | 1 |
| Country | 1 | 74 | 3 | 4 | 0.2 | 0.1 | 5 | 12 |  |
| Portugal (n=88) |  |  |  |  |  |  |  |  |  |
| Study |  | 84 | 1 |  |  |  | 1 | 9 | 5 |
| Country | 3 | 81 |  | 1 | 1 | 1 | 6 | 7 |  |
| Sweden (n=436) |  |  |  |  |  |  |  |  |  |
| Study | 38 | 2 | 0.7 | 2 |  | 0.2 | 5 | 48 | 5 |
| Country | 63 | 1 |  | 1 | 0.5 | 0.1 | 4 | 28 |  |
| The Netherlands (n=530) |  |  |  |  |  |  |  |  |  |
| Study | 21 | 10 |  | 1 | 0.2 |  | 6 | 57 | 5 |
| Country | 15 | 22 |  | 5 | 0.4 | 0.1 | 6 | 52 |  |
| United Kingdom (n=119) |  |  |  |  |  |  |  |  |  |
| Study | 13 | 29 | 0.8 | 3 |  |  | 16 | 34 | 3 |
| Country | 47 | 10 |  | 5 |  | 0.3 | 9 | 25 |  |
| USA (n=155) |  |  |  |  |  |  |  |  |  |
| Study | 17 | 29 | 0.7 | 1 | 1 | 4 | 23 | 19 | 5 |
| Country | 43 | 20 | 0.1 | 1 | 1 | 2 | 9 | 23 |  |

1. Country data has been retrieved in 2023 from a number of different online sources such as country statistics, Wikipedia information etc. and as such only provide a very broad overview.
