# Supplementary material for: Perceptions of ethical decision-making climate among clinicians working in European and US ICUs: differences between religious and non-religious healthcare professionals
Source: BMC Med Ethics. 2025 Feb 5;26:21. doi: 10.1186/s12910-025-01178-5 (PMC11796059; doi:10.1186/s12910-025-01178-5)
Supplement: Supplementary file 2 — Supplementary Material 2 [file 12910_2025_1178_MOESM2_ESM.pdf]

The DISPROPRICUS Study: Questionnaire related to the HEALTHCARE PROVIDER

Goal of the study

Clinicians perceive the care they provide as inappropriate when they feel that it clashes with their personal beliefs and/or professional knowledge. **Moral distress occurs when one feels what is the right thing to do, but institutional or other constraints make it difficult to pursue the desired course of action.** ICU workers who experience acute moral distress are at increased risk for burnout.

This study focuses on disproportionate care defined as care which is perceived as disproportionate (too much or too little) in relation to the expected prognosis of the patient in terms of expected survival or quality of life or patient or family wishes.

The goal of this study is (1) to evaluate how frequently ICU healthcare providers feel that patient care is disproportionate and (2) to identify workenvironmental factors that promote ICU workers' well-being.

Practical considerations

This survey deals with your personal characteristics and your work environment. We ask you to fill it out once at the start of the study. This questionnaire takes maximum 20 minutes to complete.

In the near future, you will be asked to shortly report about your perceptions about the appropriateness of care given to each patient who is under your care during a month span.

This is an anonymous questionnaire survey, meaning that the results will be revealed in a way that precludes identification of the participating ICUs and healthcare providers.

If you have any questions, please contact the local investigator.

Thank you for participating in this study!

Personal characteristics and working conditions

ICU number:	…..
ICU healthcare provider number:	…..

Personal characteristics

PC1. What is your age?			…. years	
PC2. What is your gender?			☐ Female	☐ Male
			1.Yes	2.No
PC3. Are you living with a partner?			☐	☐
PC4. Do you have any children?			☐	☐
PC5. What is your religion?<br>a. Roman Catholic<br>b. Protestant<br>c. Greek-orthodox<br>d. Muslim<br>e. Jewish<br>f. Buddhist<br>g. Non-religious<br>h. I do not wish to answer this question				
	1.Not important	2.Not very important	3.Import ant	4.Very Important
PC6. How important is your religion for your professional attitude towards end-of-life decisions?	☐	☐	☐	☐

Working conditions

WC1. What is your role in the ICU? (choose 1)		
a. Nurse (and/or nurse assistant >> only in the French questionnaires)	☐	
b. Head nurse	☐	
c. Junior physician (in training)	☐	
d. Senior physician	☐	
e. Head of ICU	☐	
	1. Yes, more than 50% of activities WITHIN the ICU	2. No, More than 50% of activities OUTSIDE the ICU
WC1b. Is more than 50% of your clinical activities within the ICU?	☐	☐
WC2. If you are a physician, what is your main basic medical specialty? (choose 1)		
a. Surgery	☐	
b. Anesthesiology	☐	
c. Pulmonology	☐	
d. Emergency Medicine	☐	
e. Internal Medicine	☐	
f. Hospitalist	☐	
g. ICU specialist	☐	
WC3. How many years have you had regular activities in the ICU?	.....	
WC4. How many hours on average do you work per week?	.....	
WC5. How many night shifts on average do you work per month (including during weekends)?	.....	
WC6. How many daytime shifts during weekends on average do you work per month?	.....	
	1. Yes	2. No
WC7. Are you doing ICU research or participating in an ICU working group within your ICU?	☐	☐
WC8. Have you ever been involved in a medico-legal claim against you, regardless of the outcome?	☐	☐

Job strain (demand, control, support) and intentional jobleave

To what extent do you agree with the following statements?	A1.Stronlgy disagree	A2. Mainly disagree	A3. Agree	A4. Strongly agree
JS_D 1. I have to work very hard.	☐	☐	☐	☐
JS_D 2. I am asked to do an excessive amount of work.	☐	☐	☐	☐
JS_D 3. I don't have enough time to get my work done.	☐	☐	☐	☐
JS_C 4. I don't have to do a lot of repetitive work.	☐	☐	☐	☐
JS_C 5. I have (a job which requires me) to be creative.	☐	☐	☐	☐
JS_C 6. I have (a job which requires me) to learn new things.	☐	☐	☐	☐
JS_C 7. I have a lot of say about what happens on my job/at my work.	☐	☐	☐	☐
JS_C 8. I have a lot of freedom to decide how I do my work.	☐	☐	☐	☐
JS_S 9. I work with helpful people.	☐	☐	☐	☐
JS_S 10. I work with people who take a personal interest in me.	☐	☐	☐	☐
JS_S 11. My supervisor is helpful.	☐	☐	☐	☐
JS_S 12. My supervisor is concerned about my welfare.	☐	☐	☐	☐
JL 13. I have thoughts about leaving my current position/job.	☐	☐	☐	☐

Interdisciplinary and safety culture

To what extent do you agree with the following statements?	1. Strongly disagree<br>2. Disagree<br>3. Neither<br>4. Agree<br>5. Strongly agree
I1. In my ICU, there are regular opportunities for open and informal dialogue between healthcare providers	
I2. In my ICU, there is regular structured and formal dialogue between the various disciplines within the team to discuss patient care.	
I3. In my ICU, we regularly reflect on the quality of care provided from the various points of view of the staff.	
I4. In my ICU, the teams are well coordinated/managed.	
I5. In my ICU, there is an open and constructive culture in the department such that criticism can be easily expressed.	
I6. In my ICU, discussions about patients lead to greater understanding and agreements.	
I7. In my ICU, I am always regarded and addressed by everyone in the team as a fully-fledged team member.	
I8. In my ICU, team members from another discipline respect my work.	
I9. In my ICU, I have confidence in the professional competence of my team members.	
I10. In my ICU, it is difficult to speak up if I perceive a problem with patient care	
I11. The culture in my ICU makes it easy to learn from the errors of others	

Leadership culture

Leadershipskills of the senior physicians in charge of daily patient care

To what extent do you agree with the following statements?	1. Never<br>2. Seldom<br>3. Occasionally<br>4. Often<br>5. Always
LC1. In my ICU, the physicians in charge let the team members know what is expected of them.	
LC 2. In my ICU, the physicians in charge make accurate and timely decisions.	
LC 3. In my ICU, the physicians in charge take full charge when emergencies arise.	
LC 4. In my ICU, the physicians in charge are hesitant about taking initiative in the group.	
LC 5. In my ICU, the physicians in charge help team members settle their differences.	
LC 6. In my ICU, my physicians in charge trust the team members to exercise good judgment.	
LC 7. In my ICU, the physicians in charge permit the team members to use their own judgment in solving problems.	
LC 8. In my ICU, the physicians in charge encourage initiative in the team members.	
LC 9. In my ICU, the physicians in charge treat all team members as their equals.	
LC 10. In my ICU, the physicians in charge abstain from explaining their actions.	
LC 11. In my ICU, the physicians in charge are well aware of their own emotions and attitudes.	
LC 12. In my ICU, the physicians in charge are well aware of their role model function.	
LC 13. In my ICU, the physicians in charge dare to show their vulnerability	

End-of-life care Climate

To what extent do you agree with the following statements?	1.Stronlgy disagree	2. Mainly disagree	3. Agree	4. Strongly agree
EC1. My colleagues understand my thoughts/feelings about difficult end-of-life decisions.	☐	☐	☐	☐
EC 2. In my ICU, different opinions and values concerning end-of-life are tolerated.	☐	☐	☐	☐
EC 3. In my ICU, we talk about moral problems.	☐	☐	☐	☐
EC 4. In my ICU, there is a structured, formal debriefing after a difficult patient care situation.	☐	☐	☐	☐
EC 5. In my ICU, nurses are present during the communication of end-of-life information to the family.	☐	☐	☐	☐
EC 6. In my ICU, nurses are involved in end-of-life decision-making.	☐	☐	☐	☐
EC 7. In my ICU, nurses and physicians collaborate well with one another during end-of-life situations.	☐	☐	☐	☐
EC 8. In my ICU, death is perceived as a treatment failure, so decisions to withdraw or withhold therapy are seldom taken.	☐	☐	☐	☐
EC 9. In my ICU, EOL decisions are frequently postponed.	☐	☐	☐	☐
EC 10. In my ICU, patients with little chance of recovery are frequently admitted.	☐	☐	☐	☐
EC 11. In my ICU, patients with little chance of recovery frequently occupy an ICU bed which other patients would benefit more from.	☐	☐	☐	☐

Personal opinions concerning ethical issues in the ICU:
Healthcare costs and end-of-life care

To what extent do you agree with the following statements?	1.Stronlgy disagree	2. Mainly disagree	3. Agree	4. Strongly agree
PO_HCC1. As a clinician. I have a responsibility to help control healthcare costs.	☐	☐	☐	☐
PO_HCC2. If a medical intervention has any chance (no matter how small) of helping the patient. it is the physician's duty to offer it.	☐	☐	☐	☐
PO_HCC3. Physicians should know the overall cost of the care they provide.	☐	☐	☐	☐

PO_EOL4. Are there situations when admission to ICU is inappropriate for a patient ACCORDING TO YOU (more than 1 answer possible)?	1. Yes, ICU inappropriate for this type of patients	2. No, ICU appropriate for this type of patients
1. The patient requires monitoring only	☐	☐
2. Patient with advanced dementia	☐	☐
3. Patient with advanced co-morbidities	☐	☐
4. Patient in persistent vegetative state.	☐	☐
5. Other (please specify in English) ______________________	☐	☐
