# Supplementary material for: Perceptions of ethical decision-making climate among clinicians working in European and US ICUs: differences between religious and non-religious healthcare professionals
Source: BMC Med Ethics. 2025 Feb 5;26:21. doi: 10.1186/s12910-025-01178-5 (PMC11796059; doi:10.1186/s12910-025-01178-5)
Supplement: Supplementary file 3 — Supplementary Material 3 [file 12910_2025_1178_MOESM3_ESM.docx]

**Table S3. Overall differences between religious and non-religious healthcare providers in seven ethical decision-making climate factors.**

|  | |  | Non-religious healthcare providers  (n = 2539) | | Religious healthcare providers (n = 453) | |  |  |  |  |  | |
| --- | --- | --- | --- | --- | --- | --- | --- | --- | --- | --- | --- | --- |
|  | |  |  |  |  |  |  |  |  |  |  | |
| Factor | |  | median | IQR | median | IQR | diff | p | adjusted median diff | adjusted p^1^ | Highest median after adjustment^2^ | |
| F1. Leadership by physicians | 0.08 | | | (-0.65;0.70) | **0.21** | **(-0.57;0.87)** | 0.13 | 0.06 | 0.07 | 0.26 | religious | |
| F2. Interdisciplinary reflection | 0.1 | | | (-0.63;0.71) | **0.06** | **(-0.65;0.73)** | 0.04 | 0.54 | 0.04 | 0.55 | religious | |
| F3. Culture of not avoiding EOL-DM | 0.05 | | | (-0.60;0.71) | **-0.08** | **(-0.70;0.59)** | 0.13 | 0.09 | 0.10 | 0.13 | religious | |
| F4. Mutual respect | 0.14 | | | (-0.48;0.52) | **0.12** | **(-0.61;0.42)** | 0.02 | 0.71 | 0.05 | 0.26 | religious | |
| F5. Active involvement nurses | 0.18 | | | (-0.55;0.60) | **-0.06** | **(-0.89;0.45)** | 0.24 | <0.001*** | 0.03 | 0.59 | religious | |
| F6. Active DM physicians | 0.19 | | | (-0.57;0.59) | **0.16** | **(-0.56;0.72)** | 0.03 | 0.64 | 0.03 | 0.54 | religious | |
| F7. Ethical awareness | 0.04 | | | (-0.43;0.50) | **-0.06** | **(-0.64;0.28)** | 0.10 | <0.01** | 0.02 | 0.45 | religious | |
| EOL-DM: End-of-life decision-making, DM: Decision-making.  1. Mixed model adjusting for country as a fixed effect and ICU as a random effect  2. Due to the adjustment for country and ICU, it is possible that adjusted differences in median are larger compared to unadjusted differences, yet the opposite is also a possibility. For clarity, the group with the largest adjusted median is therefore explicitly mentioned | | | | | | | | | | | |  |
| ** Significant at <0.01 level  *** Significant at <0.001 level | |  |  |  |  |  |  |  |  |  |  |  |
